# Sulforaphane-Enriched Broccoli Sprouts Pretreated by Pulsed Electric Fields Reduces Neuroinflammation and Ameliorates Scopolamine-Induced Amnesia in Mouse Brain through Its Antioxidant Ability via Nrf2-HO-1 Activation

**DOI:** 10.1155/2019/3549274

**Published:** 2019-03-27

**Authors:** Lalita Subedi, KyoHee Cho, Yong Un Park, Hyuk Joon Choi, Sun Yeou Kim

**Affiliations:** ^1^Laboratory of Pharmacognosy, College of Pharmacy, Gachon University, #191, Hambakmoero, Yeonsu-gu, Incheon 21936, Republic of Korea; ^2^BK Bio Co. Ltd., 2706-38, Iljudong-ro, Gujwa-eup, Jeju-si, Jeju-do, Republic of Korea

## Abstract

Activated microglia-mediated neuroinflammation plays a key pathogenic role in neurodegenerative diseases, such as Alzheimer's disease, Parkinson's disease, multiple sclerosis, and ischemia. Sulforaphane is an active compound produced after conversion of glucoraphanin by the myrosinase enzyme in broccoli (*Brassica oleracea* var) sprouts. Dietary broccoli extract as well as sulforaphane has previously known to mitigate inflammatory conditions in aged models involving microglial activation. Here, we produced sulforaphane-enriched broccoli sprouts through the pretreatment of pulsed electric fields in order to trigger the biological role of normal broccoli against lipopolysaccharide-activated microglia. The sulforaphane-enriched broccoli sprouts showed excellent potency against neuroinflammation conditions, as evidenced by its protective effects in both 6 and 24 h of microglial activation *in vitro*. We further postulated the underlying mechanism of action of sulforaphane in broccoli sprouts, which was the inhibition of an inflammatory cascade *via* the downregulation of mitogen-activated protein kinase (MAPK) signaling. Simultaneously, sulforaphane-enriched broccoli sprouts inhibited the LPS-induced activation of the NF-*κ*B signaling pathway and the secretions of inflammatory proteins (iNOS, COX-2, TNF-*α*, IL-6, IL-1*β*, PGE2, etc.), which are responsible for the inflammatory cascades in both acute and chronic inflammation. It also upregulated the expression of Nrf2 and HO-1 in normal and activated microglia followed by the lowered neuronal apoptosis induced by activated microglia. Based on these results, it may exhibit anti-inflammatory effects via the NF-*κ*B and Nrf2 pathways. Interestingly, sulforaphane-enriched broccoli sprouts improved the scopolamine-induced memory impairment in mice through Nrf2 activation, inhibiting neuronal apoptosis particularly through inhibition of caspase-3 activation which could lead to the neuroprotection against neurodegenerative disorders. The present study suggests that sulforaphane-enriched broccoli sprouts might be a potential nutraceutical with antineuroinflammatory and neuroprotective activities.

## 1. Introduction

Neuroinflammation plays a key role in the regulation of aging, Alzheimer's disease (AD), Parkinson's disease (PD), Huntington's disease (HD), multiple sclerosis (MS), stroke, depression, dementia, and metabolic disorders such as hypertension and diabetes [1]. Neuroinflammation is also the pathogenic hallmark of aging-related neurodegenerative conditions [1, 2]. Therefore, anti-inflammatory strategies could be efficient prophylactic and therapeutic management strategies for a number of central nervous system (CNS) disorders [3, 4]. CNS disorders may develop owing to chronic microglial activation. Glial cells, particularly microglia, are immune cells in the CNS that are responsible for the maintenance of normal homeostasis as well as repair after injury in the brain [5]. Although activated microglia are required for host defense and debris clearance in the brain, chronic microglial activation is toxic to the CNS [6]. Conversion of normal microglia to the toxic microglial phenotype (known as the M1 phenotype) is responsible for the initiation of inflammatory cascades in the CNS, particularly the production of reactive oxygen species (ROS), nitric oxide (NO), proteases, arachidonic acids, excitatory amino acids, and cytokines [7]. These neurotoxic substances trigger the oxidative stress and are responsible for the disruption of the architecture and functions of neurons, consequently leading to synaptic degeneration and neurodegeneration [8]. These neuroinflammatory and neurotoxic cascades are responsible for the hippocampal neuronal damage leading to cognitive dysfunction [9]. Inhibition of the neuroinflammation or activation of the endogenous antioxidant system might be a better alternative for the repairment of this damage. Particularly, activation of the Nrf2 and its mediated antioxidant enzyme, HO-1, can not only inhibit the inflammatory cascades but also increase the neuronal survival and hippocampal neurogenesis [10]. Extensive neuropharmacology research has succeeded in finding novel drug candidates for the treatment of CNS disorders. However, they have failed to prove their efficacy in human biological systems during clinical trials. Potential reasons for these failures were suggested to be differences in tissue physiology of the CNS, unstable pharmacokinetics, or difficulty in crossing the blood-brain barrier (BBB) [11, 12]. Given the lack of proper allopathic medications to treat neuroinflammatory disorders, there is a growing interest in complementary and alternative medication, including nutraceuticals. Consumption of dietary nutraceuticals with neuroprotection could prevent CNS diseases, overcoming the limitations of allopathic drug delivery to the CNS.

Previous reports highlight the beneficial effects of broccoli and its active compound, sulforaphane, in neurodegenerative disorders. Although broccoli has a mild effect against age-related neuroinflammation, it does not show a significant effect against lipopolysaccharide- (LPS-) induced inflammatory conditions [13]. Another independent study has revealed that sulforaphane can reverse the hyperammonemia-induced glial activation, neuroinflammation, and disturbances in neurotransmitter receptors in the hippocampus that impaired spatial learning [14]. The anti-inflammatory effect of sulforaphane has also been previously reported in rat primary microglia [15]. These previous studies suggest that bioconversion of glucoraphanin in the broccoli may increase the yield of sulforaphane. It could lead to better health benefits for patients with neuroinflammatory disorders. Either the induction of myrosin production or the increase in myrosinase activity can increase the bioconversion of glucoraphanin into sulforaphane [16]. The presence of some other proteins, such as epithiospecifier protein (ESP), can also convert glucoraphanin to sulforaphane nitrile, without affecting sulforaphane's anti-inflammatory effects [17]. An increase in myrosinase activity with reduced ESP activity can selectively enhance sulforaphane yield in the broccoli sprout, making it a better candidate for the treatment of several pathological conditions, including cancer, inflammation, neurodegeneration, and aging [18]. Pharmacokinetic studies have revealed that, in mice, sulforaphane has good absorption and distribution patterns, in various tissues of the body, including the brain, lungs, heart, liver, kidneys, and muscles, after oral administration [19]. Previous reports suggest that sulforaphane and its metabolites do not readily cross the BBB [20]. However, the disruption of the BBB during neuroinflammation and neurodegenerative conditions allows sulforaphane to permeate the BBB and enhances its anti-inflammatory effects along the brain axis [20].

Former reports have revealed that activation of myrosinase in steamed broccoli sprouts can enhance the yield of sulforaphane [20]. Similarly, pulsed electric field (PEF) pretreatment enhances the production of glucosinolate in the broccoli flower and stalk. It also increased anthocyanin's production in red cabbage [21, 22]. Thus, we postulated that PEF might also be effective in enhancing the production of sulforaphane in broccoli sprouts.

In the current study, we are to reveal whether PEF treatment can increase the sulforaphane yield in broccoli sprouts or not. We also try to figure out the difference in the biological activity of broccoli sprout before and after PEF treatment. To determine the molecular mechanism underlying the sulforaphane-enriched broccoli sprout-mediated neuroprotective effect, we assessed the effect of sulforaphane-enriched broccoli sprouts on lipopolysaccharide- (LPS-) induced proinflammatory responses in both acute and chronic microglial activation *in vitro*. *In vivo* study will be done whether sulforaphane-enriched broccoli sprouts improve the scopolamine-induced memory impairment in mice or not.

## 2. Materials and Methods

### 2.1. Reagents

The pulsed electric field (PEF) was purchased from HVP 5 (Elea, DIL, Quakenbrueck, Germany). Lipopolysaccharide (LPS) and sulforaphane were purchased from Sigma Chemical (St. Louis, MO). Dulbecco's modified Eagle medium (DMEM), Fetal bovine serum (FBS), and penicillin-streptomycin were purchased from Invitrogen (Carlsbad, CA, USA). Antibodies for cyclooxygenase 2 (COX-2), *β*-actin, GAPDH, and histone-3 were obtained from Santa Cruz Biotechnology (Dallas, Texas, United States). The antibody of inducible nitric oxide synthase (iNOS) was purchased from Abcam (Cambridge, UK). The primary antibody for *α*-tubulin, C-Jun N-terminal kinase (JNK), p38, extracellular signal-regulated kinases (ERK), NF-*κ*B, I-*κ*B, pI-*κ*B, C-Jun, pC-Jun, C-Fos, and pC-Fos were purchased from Cell Signaling (Beverly, MA, USA). Various ELISA kits like interleukin-6 (IL-6), prostaglandin E2 (PGE2), tumor necrosis factor alpha (TNF-*α*), and interleukin-*β* (IL-1*β*) were acquired from R&D Systems (Minneapolis, MN, USA).

### 2.2. Animals

**Scheme 1:**
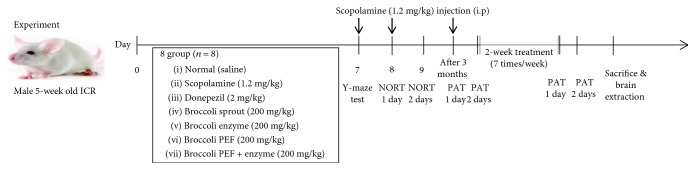
Experimental design for the *in vivo* experiment.

Male ICR mice (6 weeks; 25-30 g) were purchased from Orient Bio, Seoul, Korea. Four or five mice were placed in each cage and acclimatized for one week in laboratory conditions with food and water ad libitum. They were maintained under temperature (23 ± 1°C) and relative humidity (60 ± 10%) conditions and in a 12/12 h light and dark cycle. Animal handling and experimental procedures were conducted in accordance with the Principles of Laboratory Animal Care (GIACUC-R2017016) and the Animal Care and Use Guidelines of Gachon University, Korea. The experimental protocol is shown in Scheme 1.

### 2.3. PEF-Broccoli Preparation and Extraction

**Scheme 2:**
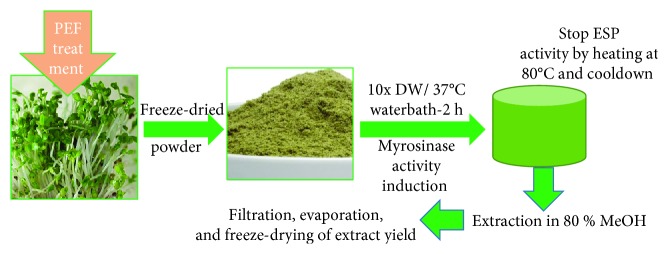
Preparation of PEF-treated broccoli sprout powder.

Broccoli sprout extraction was performed according to a previous study with slight modifications [23]. Each whole broccoli sprout (SB) as well as the hypocotyl (H), cotyledon (C), and radicle (R) were treated with PEF, at 0–7 kJ, for 3 s each, followed by freeze-drying and grinding to make fine powder ready for myrosinase activity induction. The powdered broccoli or its parts were incubated with 10x volume of distilled water, and the suspension was kept at 37°C for a total of 2 h for the induction of myrosinase activity, yielding a greater amount of glucoraphanin to sulforaphane as a final product. During this conversion, sulforaphane nitrile could also be formed with the help of epithiospecifier protein (ESP). This conversion was prevented by placing/heating the mixture at 80°C for 10 min. The mixture was cooled down by keeping it in the ice. As a final step, the suspension was mixed with methanol making the solvent as 70% methanol in total and the extraction was performed. Extraction was performed with continuous stirring and sonication for about 3 h in total. We used each sample as the following; broccoli sprout cotyledons (C) or sprout cotyledons with both PEF and myrosinase activity (C-P) and hypocotyls from broccoli sprouts (H) or hypocotyls from broccoli sprouts treated with both PEF and myrosinase activity (H-P). The supernatant was filtered (HYUNDAI Micro No. 20 filter paper, Korea) and evaporated using a rotary evaporator to remove the methanol. The extract was free-dried, and the lyophilized powder was used for subsequent experiments. Preparation of PEF-broccoli samples is shown in Scheme 2.

### 2.4. Broccoli Extract HPLC Standardization

High-performance liquid chromatography (HPLC) analysis was performed to measure the sulforaphane content in the broccoli sprout extract. The HPLC analysis was performed using a Waters system (Waters Corp., Milford, MA), consisting of a separation module (e2695) with an integrated column heater, autosampler, and photodiode array detector (2998). The UV absorbance was monitored at 200–400 nm. Quantification was conducted by integrating the peak areas at 235 nm. The injection volume was 10 *μ*L. A column (250 × 4.6 mm; particle size, 5 *μ*m; Phenomenex, USA) was installed in a column oven and maintained at 25°C. The mobile phase was composed of water containing 1% acetic acid (solvent A) and acetonitrile (solvent B). The flow rate was 0.5 mL/min. The gradient was 0.0 min, 10% B; 5 min, 20% B; 15.0 min, 40% B; 25.0 min, 60% B; 35.0 min, 90% B; and 40.0 min, 10% B. The re-equilibration time between runs was 20 min.

### 2.5. Cell Culture

The murine microglia cell (BV2) was used as a representative cell of brain microglia. The neuroblastoma cell (N2a) was used as a representative cell line of the neuron [24]. BV2 cells were kindly obtained by Dr. E. Choi at Korea University (Seoul, Korea), while N2a cells were obtained from the Korean Cell Line Bank (Seoul, Korea). BV2 & N2a cells were maintained in high-glucose DMEM supplemented with 10% heat-inactivated FBS and 1% penicillin (1 × 10^5^ U/L) and streptomycin (100 mg/L), in a humidified incubator with 5% CO_2_ at 37°C.

### 2.6. Cell Treatment and Cytotoxicity Assay

Cytotoxicity of the samples was evaluated through 3-(4,5-dimethylthiazol-2-yl)-2,5-diphenyltetrazolium bromide (MTT) assay also called the cell viability assay [25]. BV2 cells were seeded in 96-well plates overnight, and they were treated (pre/post) with different concentrations of broccoli extract with or without LPS. LPS (100 ng/mL) was added 30 min after sample treatment in case of pretreatment of prophylactic treatment condition. LPS activation in the seeded cells occurred 30 min before the sample treatment in case of posttreatment/therapeutic treatment strategy. Cells were incubated for 24 h after LPS activation, for the nitrite and cell viability assays. In case of neuronal cell viability, conditioned media from 24 h treated BV2 cells were transferred to the seeded N2a cells and incubated for another 24 h as described previously [26]. Treated cells were incubated for an hour with the MTT solution of 0.5 mg/mL concentration that will stain the viable cells into a blue color. The MTT solution was suctioned out, and 200 *μ*L of dimethyl sulfoxide was added to each well that converts the blue-stained cells to a purple-colored solution. The optical density (OD) was measured using a plate reader at 570 nm. The results were expressed as a percentage of the LPS-treated cells (LPS-treated group).

### 2.7. Nitric Oxide (NO) Measurement

Nitric oxide production inhibition by the treatment of broccoli extract and sulforaphane on LPS-stimulated BV2 cells was performed through Griess assay as described previously with slight modification [27]. BV2 cells were seeded in a 96-well plate (4 × 10^4^ cells/well) and activated with 100 ng/mL LPS, in the presence or absence of different concentrations of broccoli extract or sulforaphane, for 24 h. The nitrite level in the culture media was measured using Griess reagent (1% sulfanilamide and 0.1% N-1-napthylethylenediamine dihydrochloride in 5% phosphoric acid). A total of 50 *μ*L of supernatant was mixed with an equal volume of Griess reagent, and OD was measured at 570 nm. NG-Mono-methyl-L-arginine (L-NMMA), a well-known nitric oxide synthase (NOS) inhibitor, was used as a positive control. The pretreatment and posttreatment conditions were performed to evaluate its effect for prophylactic and therapeutic purposes.

### 2.8. Measurement of TNF-*α*, IL-1*β*, IL-6, and PGE2 Production

Cells were plated in a 6-well plate at a density of 1.5 × 10^6^ cells/well in DMEM and incubated for 24 h, to measure TNF-*α*, IL-1*β*, PGE2, and IL-6 production. The cultures were prepared and stimulated with LPS in the presence or absence of sample. After a 24 h incubation, the supernatant from the culture medium was harvested. The levels of PGE2, TNF-*α*, IL-1*β*, and IL-6 were measured. PGE2 was measured using a competitive enzyme immunoassay kit (Cayman Chemical, Ann Arbor, MI, USA), and TNF-*α*, IL-1*β*, and IL-6 were measured using ELISA development kits (R&D Systems, Minneapolis, MN, USA). The % of CV was set below 10 for every ELISA assay.

### 2.9. NF-*κ*B Assay

Nuclear and cytosolic extracts from treated microglial cells were prepared using a Nuclear/Cytosolic Extraction Kit (Active Motif, Carlsbad, CA) according to the manufacturer's protocol. Protein levels of NF-*κ*B, histone-3, I-*κ*B, and pI-*κ*B were determined using western blot analysis. Expression of nucleolic and cytosolic NF-*κ*B was measured using histone-3 and *β*-actin as loading controls, respectively. The expressions of I-*κ*B and pI-*κ*B in the cytosolic fraction were observed. The absence of *β*-actin expression in the neucleolic fraction suggested the clear separation of the neucleolic and cytosolic fractions during fractionation, without any contamination. Densitometry analysis of the bands was performed using ImageMaster™ 2D Elite software (version 3.1, Amersham Pharmacia Biotech, Buckinghamshire, UK).

### 2.10. Western Blot Analysis

Western blot analysis was conducted as previously described [26], with slight modification. Proteins obtained from BV2 cells (6 × 10^5^ cells/well), which were seeded in a 6-well plate, were used for western blot analysis. Total proteins (30 *μ*g) from each group were separated by 10% SDS-PAGE gel electrophoresis, transferred to nitrocellulose membranes, and incubated with primary antibodies against tubulin, iNOS, COX-2, ERK, pERK, JNK, pJNK, p38, pp38, NF-*κ*B, histone-3, *β*-actin, I-*κ*B, pI-*κ*B, C-Fos, pC-Fos, C-Jun, pC-Jun, and *α*-tubulin. Membranes were incubated with horseradish peroxidase-conjugated secondary antibodies, and protein bands were visualized using ECL Western Blotting Detection Reagents (Amersham Pharmacia Biotech). Densitometry analysis of the bands was performed using ImageMaster™ 2D Elite software (version 3.1, Amersham Pharmacia Biotech).

### 2.11. Y-Maze Test

The Y-maze test was performed to evaluate the spatial memory or perception of the mice after scopolamine-induced neuronal injury. The Y-shaped maze having 5 : 20 : 10 of width : length : height was prepared, and the three arms of Y shape were allocated as A, B, and C. Mice were trained in the Y-maze before the start of the experiment. Mice were randomly divided into groups of saline, donepezil (2 mg/kg, p.o.), and different extracts of the broccoli sprout (200 mg/kg, p.o.). Mice for the donepezil and extract-administered groups were challenged with scopolamine (1.2 mg/kg, i.p.) 30 min after drug administration. Donepezil is a well-known acetylcholine esterase inhibitor and is widely used as a positive control for the scopolamine-induced memory impairment model [28, 29]. Scopolamine-administered and sample treated mice were kept in the center of the maze, and the mice were allowed to enter into the arms of the maze. Mice were let to habituate for 2 min, and their entry in the arms for 8 min was evaluated. Mouse entry is set when the mouse body (from nose to tail) was fully entered into the arms. Only the entry of the mouse in all the 3 different arms consecutively was assigned with point 1 for each arm entered. Alternation behavior was defined as 3 consecutive entries into 3 different arms of the maze. Spatial perception ability was calculated according to the formula below [30].

(1) $$\begin{array}{c} \text{Voluntary alternation behavior rate }\left(\%\right)=\frac{N_{\text{alteration}}}{N_{\text{entries}}-2}\times 100, \end{array}$$

where *N*_alterations_ is the number of times alternation behavior was observed (scored by points), and *N*_entries_ is the total number of arm entries.

### 2.12. Novel-Object Recognition Test (NORT)

In order to determine the role of the broccoli sprout extract enriched with sulforaphane in memory boost, a novel object recognition test (NORT) was performed. The mouse was placed into a 45 cm × 45 cm × 50 cm box containing the novel object for about 5 minutes to habituate in the testing environment. Following the habituation, response of the mouse (time) for object recognition was recorded for 3 minutes. On the third day of testing, 1 of the objects was replaced with a new object, and the response time for the new object recognition was recorded. The concentration of mouse to recognize the new object was evaluated by the recognition index (%) as follows:

(2) $$\begin{array}{c} \text{Recognition index}\left(\%\right)=\left[\left.\text{time novel}/\text{time novel}+\text{time familiar}\right)\right]\times 100. \end{array}$$

Here, time novel is the time spent exploring the novel object, and time familiar is the time spent exploring the familiar object.

### 2.13. Passive Avoidance Task

A passive avoidance test was performed using identical boxes which are illuminated or nonilluminated of the size (20 × 20 × 20 cm), separated by a guillotine door (5 × 5 cm) as described elsewhere [31]. The illuminated compartment contained a 50 W bulb, and the floor of the nonilluminated compartment (20 × 20 × 20 cm) was composed of 2 mm stainless steel rods spaced 1 cm apart. For an acquisition trial, mice were placed in the illuminated compartment and, after 10 s, the door between the two compartments was opened. When mice entered the dark compartment, the door automatically closed and an electric foot shock (0.25 mA) was delivered for 3 s through the stainless steel rods. Twenty-four hours after the acquisition trial, the retrieval trial was conducted by placing the mice in the illuminated compartment. The latency to enter the dark compartment after opening the door was recorded. Cut-off latency was set at 600 s to avoid ceiling effects.

### 2.14. Acetylcholinesterase Activity Assay

Colorimetric assay was performed to determine the acetylcholinesterase activity using acetylthiocholine enzyme and acetylthiocholine iodide substrate as described previously [32], with slight modifications. The mouse brains were quickly harvested after CO_2_ euthanasia and well homogenized in a homogenization buffer (12.5 mM sodium phosphate buffer, pH 7.0, 400 mM NaCl) using a glass Teflon homogenizer (EYELA, Japan), and the supernatant was used for the acetylthiocholine activity assay as described previously [32]. In brief, 0.02% Tanshinone congeners were prepared in dimethyl sulfoxide and mixed with acetylthiocholine iodide solution (75 mM), buffered Ellman's reagent (10 mM), 5,5′-dithio-bis(2-nitrobenzoic acid), and 15 mM sodium bicarbonate. The total mixture was incubated for 30 min for reaction at room temperature. Absorbance was measured at 410 nm immediately after adding the enzyme source to the reaction mixtures, and readings were taken at 30 s intervals for 5 min. Donepezil was used as a positive control.

### 2.15. Data and Statistical Analysis

All results are expressed as mean ± standard error of the mean (SEM). Statistical significance between experimental groups was determined by using one-way analysis of variance (ANOVA) followed by the Tukey post hoc test using GraphPad Prism 5 (GraphPad Software Inc., La Jolla, CA, USA). Statistical significance was set at *P* < 0.05. Each experiment was performed in triplicate.

## 3. Results

### 3.1. PEF Exposure Enriched Sulforaphane Content in Broccoli Sprouts

**Figure 1:**
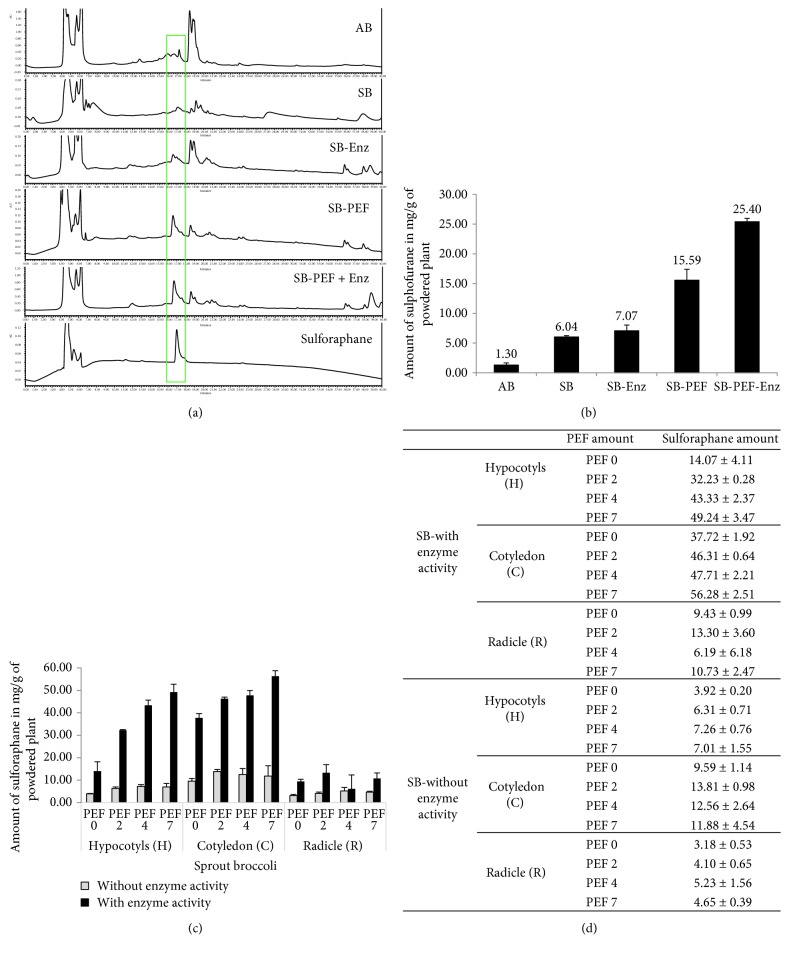
Pulsed electric field (PEF) and enzyme activity increased sulforaphane content in broccoli sprouts. Broccoli sprout was treated with PEF and extracted followed by the treatment of myrosinase for increased enzyme activity to convert glucoraphane to sulforaphane. High-performance liquid chromatography (HPLC) evaluation of different broccoli extracts was performed. (a, b) Quantitative evaluations of sulforaphane in adult broccoli (AB), broccoli sprouts (SB), broccoli and induced enzyme activity (SB-Enz), sprout broccoli with PEF (SB-PEF), and sprout broccoli with PEF and induced enzyme activity (SB-PEF + Enz), and pure sulforaphane compound samples, using HPLC. (c, d) Amount of sulforaphane in broccoli plants, particularly in the hypocotyls, cotyledons, or radicle, following different PEF concentrations, with or without enzyme activity. All data are presented as mean ± standard error of the mean of three independent experiments.

We confirmed the content of sulforaphane as a surrogate marker, using HPLC, for the quality control of broccoli sprouts.  Figure 1 shows the chromatogram of broccoli sprouts compared to that of sulforaphane. The linearity of the compound was calculated using five concentrations. The sulforaphane content of adult broccoli is found to be almost 4.5-fold lesser than that of the broccoli sprouts. Enzymatic activation of broccoli sprouts increased the sulforaphane content almost 1.2-fold more than that of the normal broccoli sprout. Sulforaphane content was increased by 2.5-fold in a broccoli sample treated with PEF only. When the enzymatically activated broccoli sprout was treated with PEF, sulforaphane yield was 4.2-fold, in particular in the cotyledon, in comparison to the broccoli sprouts. Our experiment revealed that the sulforaphane content of broccoli sprouts, particularly in the hypocotyls and cotyledons, was increased to the highest extent with the combined treatment of PEF and enhanced enzymatic activity. The radicle of the broccoli had the lowest amount of sulforaphane, which was not altered, even with exposure to PEF and enzymatic activity. Compared to broccoli sprouts, adult broccoli contains less amount of sulforaphane, which was increased with activation of enzymatic activity, PEF exposure, and the combination of both conditions as shown in Figure 1. Overall, increased enzymatic activity and PEF exposure showed a synergistic effect in upregulating the amount of sulforaphane in broccoli sprouts.

### 3.2. Broccoli Sprouts Exposed to PEF Inhibited Nitrite Production in LPS-Activated Microglia

**Figure 2:**
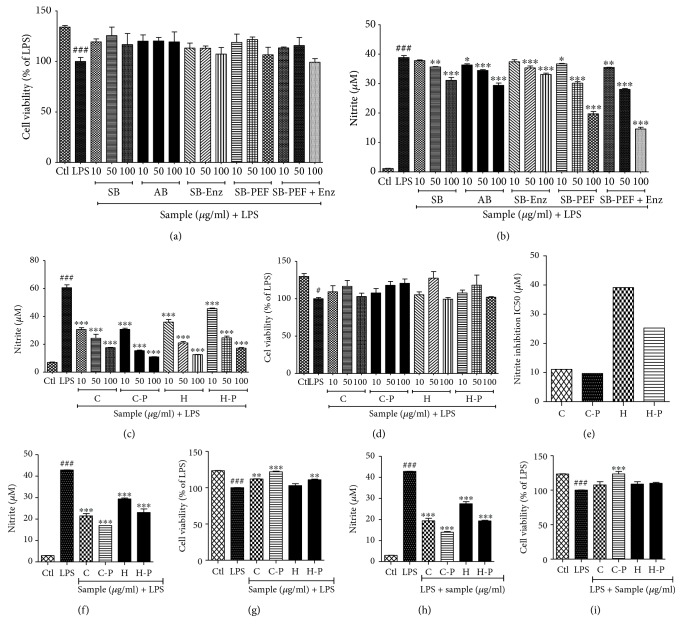
Pulsed electric field (PEF) and enzyme activity-treated broccoli sprouts inhibited nitrite production in lipopolysaccharide- (LPS-) activated BV2 microglial cells. BV2 microglial cells were pretreated with broccoli extracts, and LPS (100 ng/mL) stimulation was performed after 30 min. (a, b) Nitrite production and cell viability after different types of broccoli sample treatment. (c, d) Nitrite production and cell viability of LPS-activated microglia following pretreatment of broccoli samples. (e) IC50 value for the nitrite inhibition by C, C-P, H, and H-P treatment in LPS-activated microglia. (f-i) Nitrite inhibition and cell viability after C, C-P, H, and H-P pre- and post-treatment in LPS-activated microglia. All data are presented as mean ± standard error of the mean of three independent experiments. ^∗^*P* < 0.05, ^∗∗^*P* < 0.01, and ^∗∗∗^*P* < 0.001 indicate significant differences compared with treatment with LPS alone, while ^#^*P* < 0.05 and ^###^*P* < 0.001 indicate the significant differences compared with an untreated control group. Ctl: control; LPS: lipopolysaccharide; SB: sprout broccoli; AB: adult broccoli; SB-Enz: broccoli sprouts with induced enzyme activity; SB-PEF: broccoli sprouts with PEF treatment; SB-PEF + Enz: broccoli sprouts with PEF treatment and induced enzyme activity; C: cotyledons; C-P: cotyledons exposed with PEF + enzyme activity; H: hypocotyls; H-P: hypocotyls exposed with PEF + enzyme activity.

The ability of young and adult broccoli to inhibit NO was more pronounced when the broccoli sprouts were exposed to PEF and enzyme activity. Broccoli plants exposed to this combination were approximately 2-fold more effective at inhibiting nitrite production, compared to those exposed to either PEF or enzymatic activity only. Compared to the hypocotyls, broccoli plant cotyledons exposed to enzyme and PEF showed the highest potency. After confirming the effect of PEF and enzyme activity on broccoli, we evaluated differences in its activity, during pre-treatment and post-treatment, on LPS-activated microglial cells. The pattern of activity, as well as the extent of potency, was similar to its effects on nitrite production during pre- and post-treatment. Broccoli sprout cotyledons (C) exposed with PEF (C-P) showed better potency in comparison to normal hypocotyls (H) or PEF-exposed hypocotyls (H-P) as shown in Figure 2. From this screening, we select 100 *μ*g/mL of the concentration of C, C-P, H, and H-P for further experiment and mechanism study.

### 3.3. Broccoli Sprouts Exposed to PEF Inhibited the Expression of iNOS and COX-2, during 6 and 24 h LPS Activation

**Figure 3:**
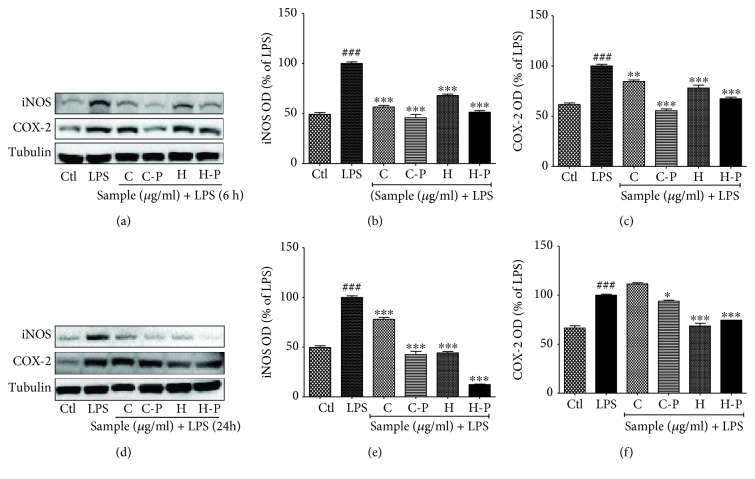
Pulsed electric field (PEF) and enzyme activity-treated broccoli sprouts inhibited expression of inducible nitric oxide synthase (iNOS) and cyclooxygenase 2 (COX-2) in lipopolysaccharide- (LPS-) activated BV2 microglial cells. BV2 microglial cells were pretreated with broccoli extracts; LPS (100 ng/mL) stimulation was performed after 30 min. (a-c) iNOS and COX-2 expression and band intensity observed in LPS-activated BV2 microglial cells after a 6 h sample treatment and LPS activation. (d-f) iNOS and COX-2 expression and band intensity observed in a 24 h sample and LPS activation in BV2 cells. All data are presented as mean ± standard error of the mean of three independent experiments. ^∗^*P* < 0.05, ^∗∗^*P* < 0.01, and ^∗∗∗^*P* < 0.001 indicate significant differences compared with treatment with LPS alone, while ^###^*P* < 0.001 indicates the significant differences compared with an untreated control group. Ctl: control; LPS: lipopolysaccharide; C: cotyledons; C-P: cotyledons exposed with PEF + enzyme activity; H: hypocotyls; H-P: hypocotyls exposed with PEF + enzyme activity.

LPS-mediated inflammation is mainly characterized by increased nitrite production and the expression of iNOS and COX-2 [27]. The expression of iNOS and COX-2 significantly increased in BV2 cells treated with LPS [26]. The PEF-enzyme activity increased the inhibitory effect of the cotyledons and hypocotyls of broccoli sprouts on the expression of these inflammatory proteins. Further, the expression of these proteins in the BV2 cells returned to almost normal levels with cotyledon and hypocotyl treatment, even in the presence of LPS in microglial cells. Sprout broccoli cotyledons (C) and cotyledon-P (C-P) were less effective at inhibiting the expression of COX-2 than that of iNOS. However, C-P and H-P significantly inhibited COX-2 expression after LPS activation as shown in Figure 3. Interestingly, normal broccoli H showed a higher potency to inhibit COX-2 expression 24 h following microglial activation. Taken together, untreated broccoli plants as well as those exposed to PEF effectively inhibited LPS-induced iNOS and COX-2 expression.

### 3.4. Broccoli Sprout Cotyledons/Hypocotyls Exposed to PEF Modulated Effector Signaling, Specifically ERK and p38 Phosphorylation

**Figure 4:**
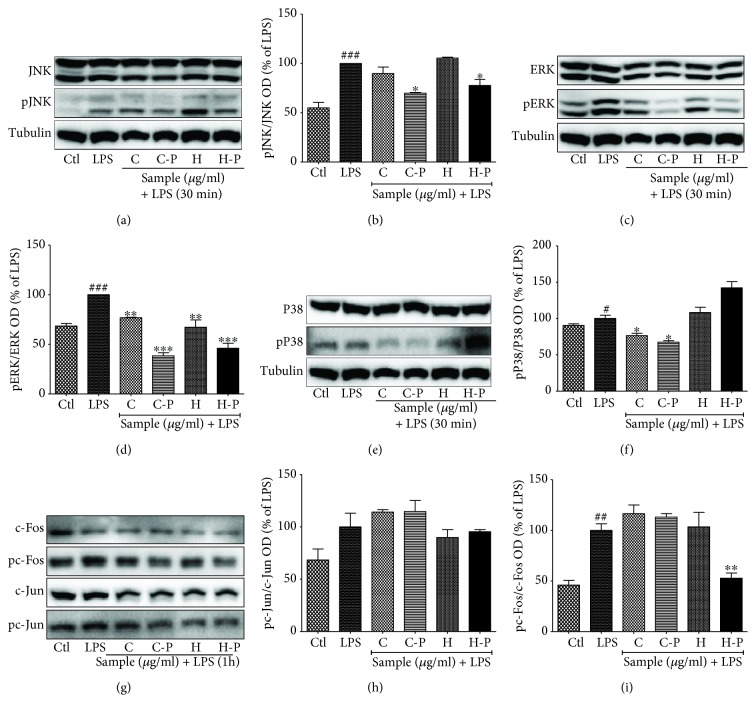
Pulsed electric field (PEF) and enzyme activity-treated broccoli sprouts modulated mitogen-activated protein kinase (MAPK) effector signaling in a 30 min lipopolysaccharide (LPS) activation. BV2 microglial cells were pretreated with broccoli extracts; LPS (100 ng/mL) stimulation was performed after 30 min. MAPK expression was measured after a 30 min LPS activation. The expression and band intensities of (a, b) JNK and pJNK, (c, d) ERK and pERK, and (e, f) p38 and pp38 in LPS-activated BV2 microglia are shown. (g-i) AP-1 signaling in LPS-activated BV2 microglia. All data are presented as mean ± standard error of the mean of three independent experiments. ^∗^*P* < 0.05, ^∗∗^*P* < 0.01, and ^∗∗∗^*P* < 0.001 indicate significant differences compared with treatment with LPS alone, while ^#^*P* < 0.05, ^##^*P* < 0.01, and ^###^*P* < 0.001 indicate significant differences compared with an untreated control group. Ctl: control; LPS: lipopolysaccharide; C: cotyledons; C-P: cotyledons exposed with PEF + enzyme activity; H: hypocotyls; H-P: hypocotyls exposed with PEF + enzyme activity.

MAPK effector signaling pathways control the production of inflammatory mediators and proinflammatory cytokines. The activation (phosphorylation) of MAPK proteins (i.e., p38, JNK, and ERK) is responsible for controlling transcription factors and ultimately the production of inflammatory mediators [33]. LPS significantly increased JNK, ERK, and p38, and this effect was inhibited by C-P. C-P inhibited the phosphorylation of ERK and p38 to an unexpectedly high extent as shown in Figure 4.

### 3.5. Broccoli Sprout Cotyledons/Hypocotyls Exposed to PEF Modulated Effector Signaling Short-Term and Chronic LPS Activation in BV2 Microglial Cells

**Figure 5:**
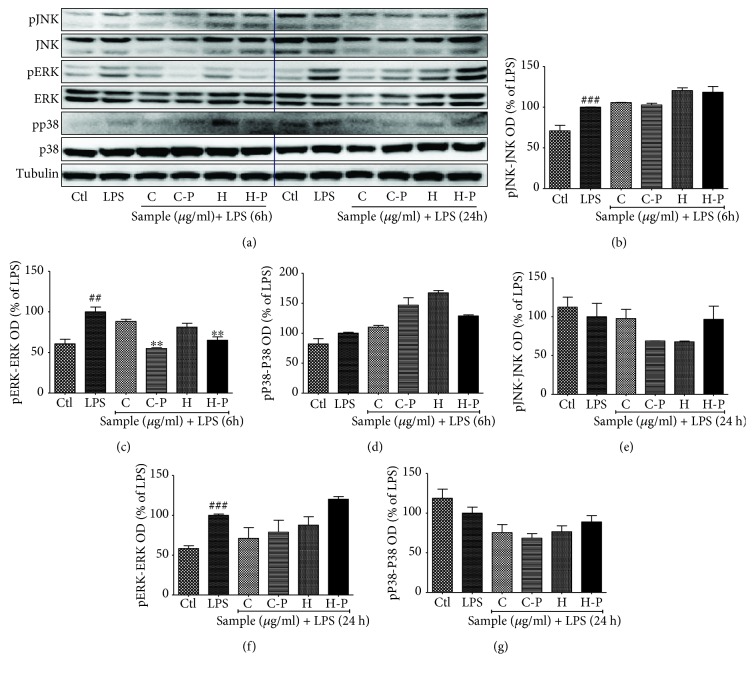
Pulsed electric field (PEF) and enzyme activity-treated broccoli sprouts modulated mitogen-activated protein kinase (MAPK) effector signaling after 6 and 24 h of lipopolysaccharide (LPS) activation. BV2 microglial cells were pretreated with broccoli extracts; LPS (100 ng/mL) stimulation was performed after 30 min. MAPK modulation was observed after 6 and 24 h of LPS activation. (a) MAPK expression in 6 h and 24 h LPS activation. (b-d) JNK and pJNK, ERK and pERK, and p38 and pp38 band intensity in 6 h LPS-activated BV2 microglia. (e-g) JNK and pJNK, ERK and pERK, and p38/pp38 band intensity in 24 h LPS-activated BV2 microglia. All data are presented as mean ± standard error of the mean of three independent experiments. ^∗^*P* < 0.05, ^∗∗^*P* < 0.01, and ^∗∗∗^*P* < 0.001 indicate significant differences compared with treatment with LPS alone, while ^#^*P* < 0.05, ^##^*P* < 0.01, and ^###^*P* < 0.001 indicate significant differences compared with an untreated control group. Ctl: control; LPS: lipopolysaccharide; C: cotyledons; C-P: cotyledons exposed with PEF + enzyme activity; H: hypocotyls; H-P: hypocotyls exposed with PEF + enzyme activity.

Both 6 h and 24 h LPS activation sufficiently activated MAPK phosphorylation, which was slightly or significantly altered by C, C-P, H, and H-P treatment. Although both C-P and H-P extensively inhibited ERK phosphorylation during the 6 h (short-term) and 24 h (chronic) LPS activation, the inhibitory effect of cotyledons was weak. H samples, however, increased ERK phosphorylation during chronic activation. The inhibitory effect of C and C-P treatment on p38 was very promising during the 6 h short-term activation and 24 h chronic activation as shown in Figure 5. Tubulin was used as a loading control for these experiments.

### 3.6. Broccoli Sprout Cotyledons Exposed to PEF Inhibited NF-*κ*B- and AP-1-Mediated Transcription of Inflammatory Proteins

**Figure 6:**
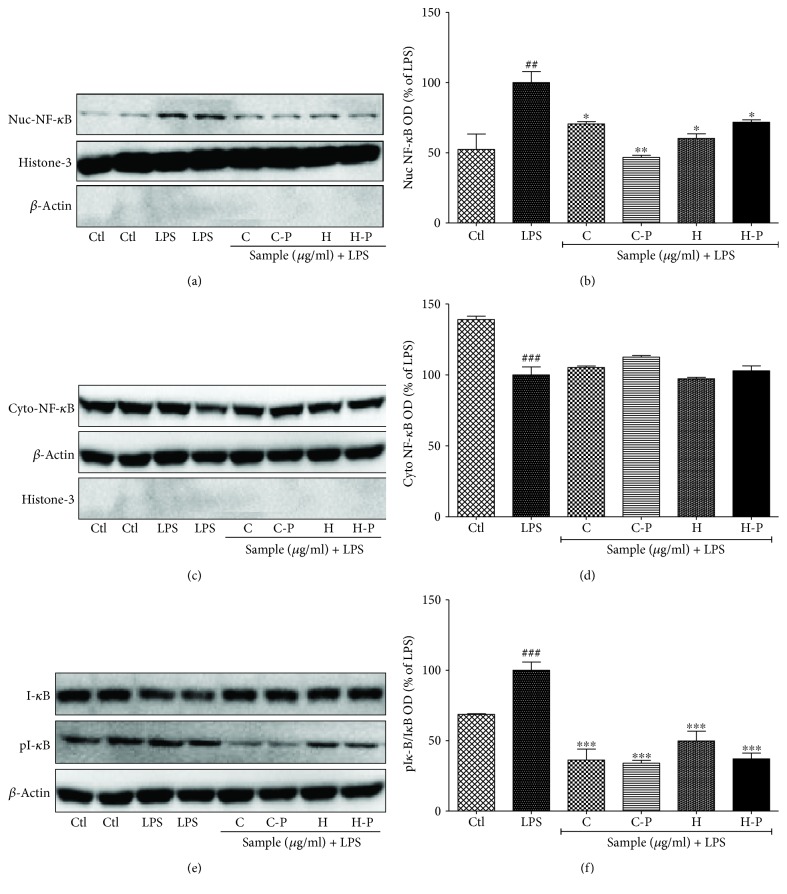
Pulsed electric field (PEF) and enzyme activity-treated broccoli sprouts inhibited NF-*κ*B translocation and I-*κ*B phosphorylation in lipopolysaccharide- (LPS-) activated BV2 cells. BV2 microglial cells were pretreated with broccoli extracts; LPS (100 ng/mL) stimulation was performed after 30 min. NF-*κ*B, I-*κ*B, and pI-*κ*B expression was observed after 1 h of LPS activation. (a, b) Nuclear NF-*κ*B expression and band intensity. Histone-3 was used as loading control. (c, d) Cytosolic NF-*κ*B expression and band intensity. *β*-Actin was used as loading control. (e, f) Cytosolic I-*κ*B and pI-*κ*B expression and band intensity. *β*-Actin was used as loading control. All data are presented as mean ± standard error of the mean of three independent experiments. ^∗^*P* < 0.05, ^∗∗^*P* < 0.01, and ^∗∗∗^*P* < 0.001 indicate significant differences compared with treatment with LPS alone, while ^#^*P* < 0.05, ^##^*P* < 0.01, and ^###^*P* < 0.001 indicate significant differences compared with an untreated control group. Ctl: control; LPS: lipopolysaccharide; C: cotyledons; C-P: cotyledons exposed with PEF + enzyme activity; H: hypocotyls; H-P: hypocotyls exposed with PEF + enzyme activity.

NF-*κ*B and AP-1 are the major transcription factors that are responsible for altering the production of inflammatory proteins and proinflammatory cytokines in LPS-activated BV2 cells. Both the cotyledons and hypocotyls of broccoli sprouts can inhibit NF-*κ*B activity; however, this effect was more prominent following exposure to PEF. LPS treatment significantly upregulated nuclear NF-*κ*B and decreased cytosolic NF-*κ*B. This effective translocation was necessary for further transcription. However, this cascade was reversed by PEF-treated broccoli sprout treatment. In addition to inhibiting NF-*κ*B translocation, broccoli C-P and H-P also inhibited the phosphorylation of I-*κ*B and increased its inactive form. Histone-3 was used as a loading control for nuclear proteins, while B-actin was used for cytosolic proteins as shown in Figure 6. No significant changes were measured in AP-1 signaling after broccoli treatment.

### 3.7. Broccoli Sprout Cotyledons Exposed to PEF Inhibited Production of Proinflammatory Cytokines and Inhibited Neuronal Death Induced by Activated Microglia

**Figure 7:**
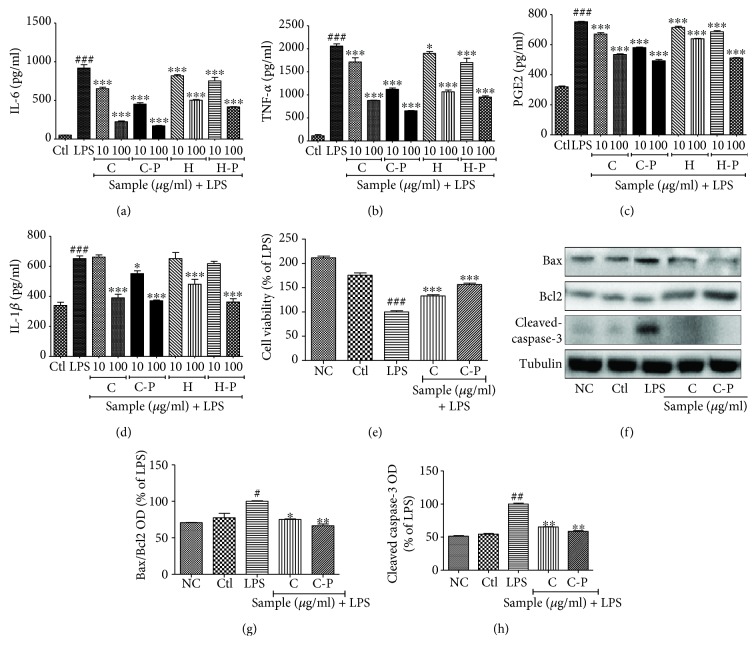
Pulsed electric field (PEF) and enzyme activity-treated broccoli sprouts inhibited proinflammatory cytokine production in LPS-activated microglia and also reduced the neuronal death caused by activated microglia. BV2 microglial cells were pretreated with broccoli extracts; LPS (100 ng/mL) stimulation was performed after 30 min. Proinflammatory cytokines were measured in the conditioned medium of the treated cells using enzyme-linked immunosorbent assays after 24 h of LPS activation. (a) Interleukin- (IL-) 6 production. (b) Tumor necrosis factor- (TNF-) *α* secretion. (c) Prostaglandin E2 (PGE2) secretion. (d) IL-1*β* secretion. Similarly treated BV2 cells in the conditioned medium were transferred to seeded N2a cells in the 6-well plate. Cell viability and proteins expression in the N2a cells were evaluated after 24 h of CM treatment. (f) N2a cell viability after activated microglial CM treatment. (f-h) Apoptosis-related protein expression in BV2 CM-treated N2a cells and their quantification. All data are presented as mean ± standard error of the mean of three independent experiments. ^∗^*P* < 0.05, ^∗∗^*P* < 0.01, and ^∗∗∗^*P* < 0.001 indicate significant differences compared with treatment with LPS alone, while ^#^*P* < 0.05, ^##^*P* < 0.01, and ^###^*P* < 0.001 indicate significant differences compared with the untreated control group. NC: normal control; Ctl: control; LPS: lipopolysaccharide; C: cotyledons; C-P: cotyledons exposed with PEF + enzyme activity; H: hypocotyls; H-P: hypocotyls exposed with PEF + enzyme activity.

Next, we evaluated the effects of broccoli cotyledons and hypocotyls on the production of inflammatory cytokines. The cotyledons and hypocotyls of normal broccoli sprouts, as well as those exposed to PEF and enzyme, significantly inhibited the production of proinflammatory cytokines, such as TNF-*α*, IL-6, IL-1*β*, and PGE2. We demonstrated that broccoli plants showed the highest effect inhibiting IL-6 and IL-1*β* production and the lowest efficacy in inhibiting TNF-*α* and PGE2 production. The low potency by that in inhibiting COX-2 production is due to weak potency in inhibiting PGE2 production as shown in Figure 7. After confirming the anti-inflammatory potential of C and C-P, we checked its role in the neuronal survival against activated microglia-induced toxicity to neurons. LPS-induced activation of the microglia resulted in the production of various inflammatory mediators which are lethal to neuronal cells, but treatment of C and C-P to the activated microglia lowered the inflammatory cascades and hence made it possible to inhibit neuronal death by lowering the Bax/Bcl2 ration and expression of cleaved caspase-3. In both of the cases, C-P showed a slightly higher potential to that of C alone. This result provides a strong cue that C and C-P might prevent neuronal death, and hence, they can prevent neurodegeneration induced either by neuroinflammation/activated microglia or by other toxicities.

### 3.8. Broccoli Samples with or without PEF Increased the Nuclear Factor Erythroid 2–Related Factor 2 (Nrf2) and Heme Oxygenase- (HO-) 1 Expression in the Normal BV2 Cells and against Scopolamine-Induced Amnesia in Mouse Brain Tissue Samples

**Figure 8:**
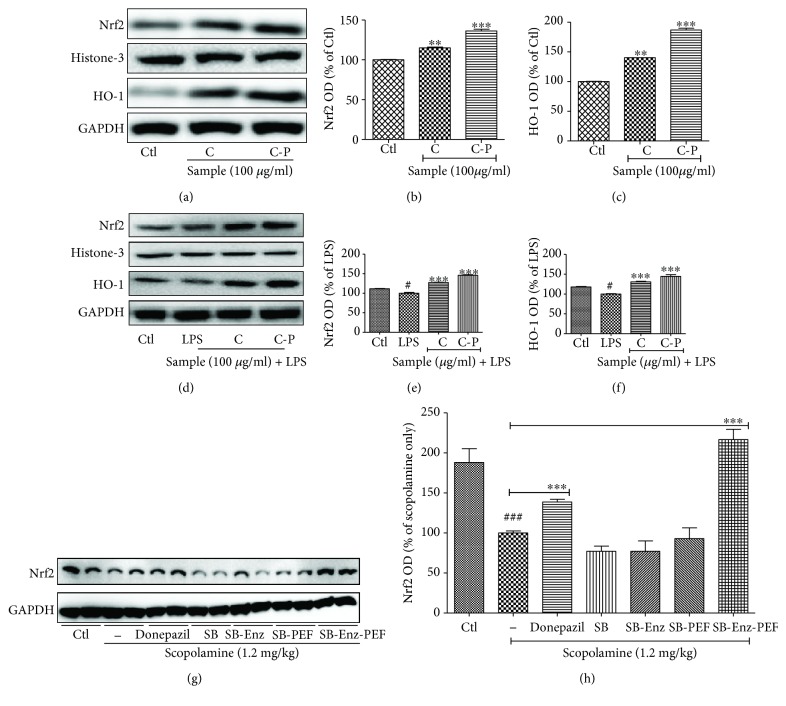
Broccoli sample treatment increased the Nrf2-HO-1 expression showing antioxidant effect in microglial cells (normal and LPS-activated conditions) as well as against a scopolamine-induced amnesia model in mice. BV2 microglial cells were treated with the broccoli sample itself. (a-c) Nrf2-HO-1 expression in normal microglia after broccoli cotyledon treatment and their quantifications. (d-f) Nrf2-HO-1 expression in LPS-activated microglia after broccoli cotyledon treatment and their quantifications. Mice were continuously exposed with scopolamine and broccoli samples for two weeks' period. Animal were sacrifices, and the brain samples were homogenized and tissue lysates were separated using the western blot technique. (g, h) Nrf2 expression and its quantification in the mouse whole brain sample. Histone-3 and GAPDH were used as a loading control for respective proteins. All data are presented as mean ± standard error of the mean of three independent experiments. ^∗^*P* < 0.05, ^∗∗^*P* < 0.01, and ^∗∗∗^*P* < 0.001 indicate significant differences compared with the untreated control group in (c, d) and only scopolamine-treated group in (f) while ^#^*P* < 0.05 and ^###^*P* < 0.001 indicate significant differences compared with the untreated control group. C: cotyledons; C-P: cotyledons exposed with PEF and enzyme activity; SB: sprout broccoli; SB-Enz: sprout broccoli with induced enzyme activity; SB-PEF: sprout broccoli with PEF treatment; SB-Enz-PEF: sprout broccoli with activated enzyme activity and PEF treatment.

Sulforaphane is known as an Nrf2 activator; in our study, we also observed that treatment of C and C-P significantly increased the expression of Nrf2 and antioxidant protein HO-1 in normal as well as LPS-activated microglia as shown in Figure 8. The increased HO-1 and Nrf2 are higher in the case of the PEF-exposed sample, and it must be because of the increased sulforaphane content in it. This suggests that the better potency of C-P to induce Nrf2/HO-1 activation in direct as well as LPS-activated microglia not only can show its antioxidant effect directly in the brain cells but also can lower the activated microglia-induced inflammatory cascades. In addition to this, SB-PEF or SB-Enz-PEF has the highest amount of sulforaphane, which is responsible for the inhibition of the neuroinflammation and neurodegeneration against scopolamine toxicity. In the animal brain, we clearly noticed that SB-Enz-PEF showed a sharp increase in Nrf2 expression (Figure 8) suggesting that increased Nrf2 was responsible for the improved memory, cognition, and increased latency time in the passive avoidance test.

### 3.9. Sprout Broccoli Samples Repair the Scopolamine-Induced Memory and Cognitive Impairment In Vivo

**Figure 9:**
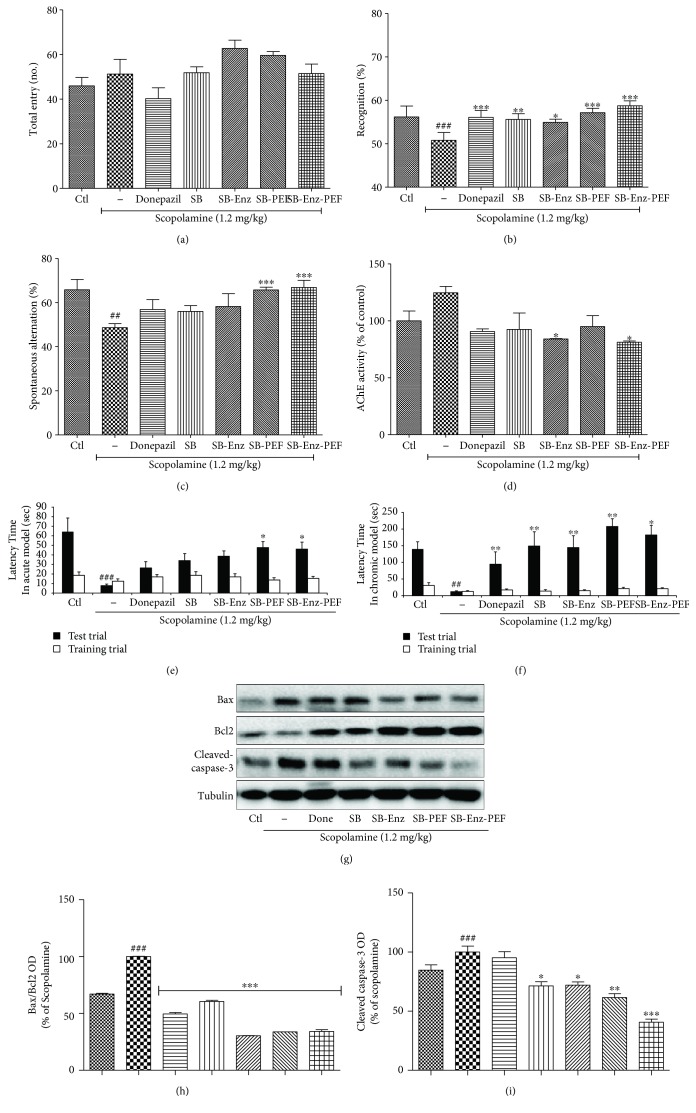
Broccoli sprout extract repaired the memory impairment by inhibiting neuronal apoptosis against scopolamine induced toxicity *in vivo*. Experimental animals were exposed with 1.2 mg/kg scopolamine and 2 mg/kg of donepezil for the donepezil group and 200 mg/kg broccoli samples. The treated animal's spatial memory was evaluated by the (a, b) Y-maze test, (c) novel object recognition test (NORT), (d) acetylcholine esterase activity assay in animal brain, (e, f) passive avoidance test in acute condition (one time per-oral treatment and toxicity induction) and chronic condition (two weeks' per-oral treatment and toxicity induction). After sacrifice, mouse whole brains were collected and homogenized with tissue lysis buffer and western blot analysis was performed. (g-i) Bax, Bcl2, and cleaved caspase-3 expression and their quantification. All data are presented as mean ± standard error of the mean of three independent experiments. ^∗^*P* < 0.05, ^∗∗^*P* < 0.01, and ^∗∗∗^*P* < 0.001 indicate significant differences compared with treatment with scopolamine alone while ^##^*P* < 0.01 and ^###^*P* < 0.001 indicate the significant differences compared with an untreated control group. Ctl: control; SB: sprout broccoli; SB-Enz: sprout broccoli with induced enzyme activity; SB-PEF: sprout broccoli with PEF treatment; SB-Enz-PEF: sprout broccoli with activated enzyme activity and PEF treatment.

Oral administration of the sprout broccoli sample 30 min before scopolamine administration significantly inhibited the spontaneous alteration almost 3-fold in comparison to the untreated control group (Figure 9). The sprout broccoli treated with PEF and sprout broccoli with enzyme activation and PEF treatment showed the highest and significant ability to repair the memory impairment. SB-PEF and SB-Enz-PEF bring the value of spontaneous alteration almost similar to that of untreated control mice. The role of SB-PEF and SB-Enz-PEF on memory impairment was reaffirmed through the NORT assay. The broccoli samples showed a significantly improved ability to recognize the novel object compared to that of scopolamine-administered mice. Interestingly, the effects of SB-Enz and SB-Enz-PEF for the novel object recognition were higher than that of the well-known positive control, donepezil. Additionally, acetylcholine esterase activity in the brain samples revealed that only the sprout broccoli with enhanced enzyme activity and SB-Enz-PEF significantly inhibited the level of AchE in the brain which might be responsible for the memory improvement against scopolamine toxicity. Beside these changes, normal/PEF broccoli further improved the cognitive ability of mice against scopolamine-induced memory impairment as evidenced by the passive avoidance test in scopolamine-induced acute and chronic models of memory impairment. Scopolamine dramatically reduced the latency time while the sprout broccoli with PEF and SB-Enz-PEF treatment remarkably recovered the latency time in the acute model. In case of chronic impairment, the broccoli samples increased the latency time suggesting their capacity to inhibit scopolamine toxicity. In both cases, SB-PEF and SB-Enz-PEF showed better potency than did the positive control donepezil (Figure 9). Donepezil and all the broccoli samples lowered the Bax/Bcl2 ration while only the broccoli samples lowered the expression of cleaved caspase-3 suggesting that the broccoli sample, especially SB-Enz-PEF, possesses higher potency to increase cell survival and decrease neuronal death in the dementia model like scopolamine treatment. Inhibition of the apoptosis-related proteins in SB-ENZ, SB-PEF, and especially SB-Enz-PEF further clarified that inhibition of neuronal death induced survival which could also take part in the higher potency to improve the cognitive function against scopolamine-induced amentia mice.

## 4. Discussion

Sulforaphane is an active compound of broccoli, *Brassica oleracea* var. italic, which is produced after conversion of glucoraphanin in the presence of the myrosinase enzyme [34]. Increased amounts of glucoraphanin or myrosinase activity can enhance sulforaphane production in broccoli or broccoli sprouts. We selected broccoli sprout for the current study since it contains more glucoraphanin than does the adult broccoli [16]. Previous studies suggested that steaming broccoli sprouts increases the enzymatic conversion of glucoraphanin to sulforaphane by lowering sulforaphane nitrile [35]. PEF pretreatment promotes the production of glucosinolate, including glucoraphanin, in broccoli flowers and stalk [21]. Thus, we exposed broccoli sprouts to PEF and myrosinase and evaluated its effect on sulforaphane production. The amount of sulforaphane was increased after enzymatic activity, which was further elevated following PEF exposure. In this study, the elevated sulforaphane content in the broccoli sprout showed better anti-inflammatory activities than did the untreated control, emphasizing the importance of enzymatic activation and PEF treatment.

Accumulating evidences regarding the anti-inflammatory potential of natural products and their isolated compounds focus on the scavenging of oxidative stress and downregulating the inflammatory cascades [36]. One of the key molecules participating in such inflammatory disease conditions, especially in neuroinflammatory disorders, is NO [37]. NO in the inflammatory condition is produced by iNOS, which mainly exaggerates oxidative stress in the neurons leading to their degeneration [38]. Inhibition of iNOS or NO production, therefore, could mitigate oxidative stress and further inflammatory cascades in neurodegenerative diseases. Previous studies reported that broccoli sprout inhibited inflammation in endothelial cells [39] and in LPS-treated mice [13]. Additionally, sulforaphane inhibited iNOS-mediated NO production in activated microglia [40]. Along with iNOS-mediated NO production, COX-2-mediated prostaglandin release is also the key pathogenic event in various inflammatory conditions [41]. Elevated expression of iNOS and COX-2 can occur with toxin exposure and in neuroinflammatory diseases. The chronic overexpression of iNOS and COX-2 is typical in AD, PD, neuropathic pain, etc., in which overexpression of these proteins mainly occurs in microglia [42, 43]. Therefore, minimizing the activation of COX in neuroinflammatory conditions is a desirable approach to achieving the neuroprotective effects. In the current study, enhanced expression of sulforaphane in broccoli sprout dramatically downregulated the COX-2-mediated PGE2 production in activated microglia. Although C-P did not significantly alter the expression of COX-2 in long-term microglial activation, its potency in inhibiting iNOS was much better in long-term LPS exposure than that in short-term microglial activation. Previously, sulforaphane is reported to downregulate COX-2 expression [44]. Our study not only confirms these existing evidences but also provides noble insights exploring that PEF treatment further increased the NO inhibiting potential of broccoli sprout through elevated sulforaphane content.

MAPK effector signaling is responsible for altering the expression and secretion of inflammatory mediators as well as proinflammatory cytokines [45]. MAPK proteins such as ERK, JNK, and p38 play essential roles in neuroinflammation [46]. P38, in particular, is responsible for the induction of apoptosis, differentiation, and regulation of inflammatory responses. LPS activates p38 downstream from TLR4 activation, promoting proinflammatory cytokines, such as TNF-*α*, IFN-*γ*, IL-1*β*, IL-12, IL-6, and IL-23, which are responsible for neuroinflammation [47, 48]. Similarly, JNK pathways can further phosphorylate c-Jun and respond to cytokines, like TNF-*α* and IL-1*β*, and growth factors [49]. The activation of JNK pathways plays a significant role in Tau pathology; therefore, inhibition of JNK/NF-*κ*B pathways is beneficial for AD and other neuroinflammatory conditions [50]. A growing body of evidences also suggests that MEK/ERK pathways are responsible for the elevated level of TNF-*α*, IL-1*β*, IL-6, and iNOS following ischemia, which further highlights their neuroinflammatory role [50]. Thus, modulation of p38, JNK, and ERK could be the potential therapeutic strategy in various inflammatory and neurodegenerative conditions in the brain. In the present study, the broccoli sprouts exposed to PEF enzyme significantly inhibited the phosphorylation of ERK, JNK, and p38, during the 30 min and 6 h LPS activation; p38 activation alone was maintained for 24 h of LPS activation. The sulforaphane-enriched broccoli did not change in the activation (phosphorylation) of c-Jun. However, H-P showed the significant inhibition of C-Fos in LPS-activated microglia. This result suggests that PEF-exposed broccoli hypocotyls might have a role in inhibiting pC-Fos-mediated signaling for inflammatory cascades.

MAPK signaling controls NF-*κ*B-mediated transcription of inflammatory mediators such as cytokines and chemokines. NF-*κ*B/AP-1 and MAPK pathways play a key role in the production of cytokines such as TNF-*α*, IL-6, and IL-8 in BV2 microglial cells [51, 52]. In our study, C-P significantly inhibited the phosphorylation of ERK, JNK, and P38 nonspecifically, during the 30 min, 6 h, and 24 h LPS-induced microglial activation. This alteration might induce the significant inhibition of nuclear NF-*κ*B while increasing cytosolic NF-*κ*B. The inhibition of NF-*κ*B-mediated transcription of inflammatory proteins was further confirmed by the increased and decreased expression of I-*κ*B and pI-*κ*B, respectively. The inhibition of MAPK-NF-*κ*B was further characterized by the significant inhibition of TNF-*α* and IL-6 production in LPS-activated BV2 microglial cells. C-P showed the highest potency for all these events, and this might be due to the higher amount of sulforaphane.

Previously, broccoli sprout extract and sulforaphane are reported as activators of well-known antioxidant molecules Nrf2/HO-1 [53]. Treatment of C and C-P activated the expression of Nrf2 and HO-1 as did by the sulforaphane treatment in normal and LPS-activated microglial cells. This effect of broccoli extract and sulforaphane can further inhibit the inflammatory cascades either through inhibiting TLR4-mediated inflammatory cascades or by their antioxidant potentials. Therefore, it is thought to be a potential mechanism that indicates the antineuroinflammatory effect of sulforaphane. Nrf2 inhibits the neuroinflammation as well as neurodegeneration against various models of brain disorders in both *in vitro* and *in vivo* experimental models [54, 55]. Previous reports suggested that sulforaphane protected neurons against rotenone-induced toxicity in an *in vivo* model which is mediated through the activation of the Nrf2 pathway [56]. In this study, SB-Enz-PEF significantly improved the scopolamine-induced spontaneous alteration as determined by the Y-maze test, novel object recognition test, and AchE activity inhibition test indicating that sulforaphane could improve memory impairment in different neurological disorders. SB-Enz-PEF also increased latency time in PAT assay. Moreover, through *in vitro* analysis, we found that the SB-Enz-PEF-treated conditioned medium from LPS-stimulated BV2 cells not only increased the neuronal cell survival but also attenuated the apoptotic proteins in neurons. Our data demonstrated that the protective effects of SB-ENZ-PEF are mediated through the Nrf2 signaling pathway; however, the overall neuroprotection of broccoli and sulforaphane might be mediated through the antiapoptotic effects, in particular downregulating caspase-3 activation. Sulforaphane-enriched broccoli extract, therefore, could mediate the neuroprotection in various neurodegenerative models, and the plausible pathway for the neuroprotection could be due to the combination of Nrf2 activation and its antiapoptotic effects.

**Figure 10:**
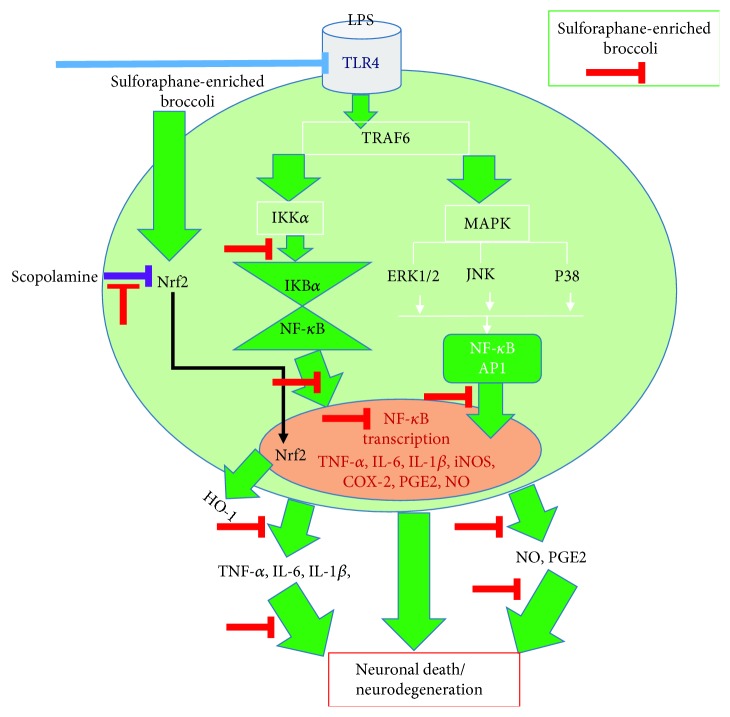
Schematic diagram for the antineuroinflammatory and antiamnesic effects of broccoli sprout extract treated with PEF.

Taken together, in this study, we provided the new insight of increased-sulforaphane-mediated protective effects in neuroinflammatory models. Being multifunctional, identification of a particular therapeutic target for phytochemicals/nutraceuticals to show desired biological activity is a key issue for the new drug development [57, 58]. However, the technique that increased the particular bioactive compounds, as PEF-induced sulforaphane in this study, could enhance the therapeutic benefit of phytochemicals. Additionally, a time point study of iNOS/COX-2 and MAPK signaling in this study could be the magnificent cues for the further *in vivo* experiment including both acute (such as ischemia, traumatic brain injury) and chronic (such as AD, PD) inflammatory conditions. Our findings demonstrated that sulforaphane-enriched broccoli sprout showed antineuroinflammatory and neuroprotective effects *in vitro* and showed the protective effects in mice against scopolamine-induced amnesia *in vivo* through Nrf2 activation. Finally, in most studies, the biological activity of sulforaphane, specifically of (−)L-isothiocyanato-4R-(methyl-sulfinyl)-butane, is demonstrated using the racemic mixture, despite the fact that humans are exposed only to the R-enantiomer through their diet. Therefore, it would be tempting to determine the role of R- and S-sulforaphane as future independent studies. The overall findings of our current study are summarized in Figure 10.

## Data Availability

All the data have been included in the manuscript and it can also be provided by the corresponding author on request.
